# Supplementary material for: Mesolimbic confidence signals guide perceptual learning in the absence of external feedback
Source: eLife. 2016 Mar 29;5:e13388. doi: 10.7554/eLife.13388 (PMC4821804; doi:10.7554/eLife.13388)
Supplement: Supplementary file 1. — DOI: http://dx.doi.org/10.7554/eLife.13388.016 [file elife-13388-supp1.docx]

**Supplementary file 1.** Model parameters. Parameter values are shown both for the group-level MLE stage and for the participant-level MLE stage (median ± SE of the median). As to be expected, the initial weight values showed a bias towards a higher value for signal weights, reflecting participants’ above-chance performance for their starting contrasts (which corresponded to 80.35% correct responses in the pre-test).

| **Parameter** | $\mathbf{w}_{\mathbf{signal}}^{\mathbf{0}}$ | $\mathbf{w}_{\mathbf{noise}}^{\mathbf{0}}$ | **α_w_** | **α_c_** | **β** | **λ** | **σ** |
| --- | --- | --- | --- | --- | --- | --- | --- |
| **Group-level** | 5.82 | 5.63 | 0.0013 | 0.384 | 13.96 | 4.92 | 0.464 |
| **Subject-level: median ±**  **SE of the median** |  |  | 0.0018 ± 0.0007 | 0.533 ± 0.077 | 17.06 ± 1.09 | 3.90 ± 0.70 | 0.314 ± 0.015 |
