# Supplementary material for: Mesolimbic confidence signals guide perceptual learning in the absence of external feedback
Source: eLife. 2016 Mar 29;5:e13388. doi: 10.7554/eLife.13388 (PMC4821804; doi:10.7554/eLife.13388)
Supplement: Supplementary file 2. — DOI: http://dx.doi.org/10.7554/eLife.13388.017 [file elife-13388-supp2.docx]

**Supplementary file 2.** List of active brain regions in the model-based fMRI analysis of confidence prediction errors (CPEs). List of brain regions exhibiting parametrically modulated activity by CPEs, family-wise error corrected at the whole-brain level. No additional brain regions beyond those reported in the main text were significantly modulated by expected confidence.

|  |  |  | **MNI coordinates** | | |  |  |  |
| --- | --- | --- | --- | --- | --- | --- | --- | --- |
|  |  | **Laterality** | **x** | **y** | **z** | **df** | **t** | **p_FWE_** |
|  |  |  |  |  |  |  |  |  |
| *Positive relationship with CPE* | |  |  |  |  |  |  |  |
|  | **Ventral striatum** | L | -16 | 8 | -10 | 28 | 7.64 | <.001 |
|  |  | R | 16 | 14 | -8 | 28 | 7.81 | <.001 |
|  | **Posterior cingulate cortex** | L | -12 | -58 | 22 | 28 | 6.23 | .024 |
|  |  | R | 8 | -62 | 24 | 28 | 8.38 | <.001 |
|  | **Thalamus** |  | 0 | -16 | 10 | 28 | 7.81 | <.001 |
|  | **Infracalcarine occipital cortex** | L | -16 | -96 | 2 | 28 | 6.59 | .011 |
|  |  | R | 20 | -96 | 6 | 28 | 6.49 | .014 |
| *Negative relationship with CPE: no significant effects* | | | | | | | | |
